# Prognostic factors for progression‐free and overall survival in malignant pleural mesothelioma

**DOI:** 10.1111/1759-7714.13814

**Published:** 2021-03-04

**Authors:** Jordi Guzmán‐Casta, Sonia Carrasco‐CaraChards, Jorge Guzmán‐Huesca, Carla Paola Sánchez‐Ríos, Rodrigo Riera‐Sala, José Fabián Martínez‐Herrera, Erika Sagrario Peña‐Mirabal, Diana Bonilla‐Molina, Jorge Arturo Alatorre‐Alexander, Luis Manuel Martínez‐Barrera, Jerónimo Rafael Rodríguez‐Cid

**Affiliations:** ^1^ Clinic of Thoracic Oncology Instituto Nacional de Enfermedades Respiratorias, Dr. Ismael Cosío Villegas Ciudad de México Mexico; ^2^ Facultad de Medicina Universidad Nacional Autónoma de México Ciudad de México Mexico; ^3^ Internal Medicine Bonita Community Health Center, Florida EE.UU. Bonita Springs Florida USA; ^4^ Department of Pathology Instituto Nacional de Enfermedades Respiratorias Ismael Cosío Villegas Mexico City Mexico; ^5^ Department of Internal Medicine Ciudad de México Hopital Ángeles Lomas Mexico

**Keywords:** Malignant pleural mesothelioma, overall survival, prognostic factors, progression‐free survival

## Abstract

**Background:**

Malignant pleural mesothelioma is an infrequent neoplasia with a poor prognosis and the majority of patients already have advanced disease at the time of presentation. Exposure to asbestos is the most important risk factor for malignant pleural mesothelioma. Mesothelioma is a neoplasia with a long preclinical stage that can span from 15 to 40 years.

**Methods:**

This was a descriptive, observational, retrospective study of 136 patients with a confirmed diagnosis of mesothelioma, which compared histological subtypes, immunohistochemical biomarkers, concomitant chronic degenerative diseases, tobacco use, age at the time of diagnosis, clinical stage and chemotherapy agents used or other treatments such as radiotherapy and surgery to identify all the factors that impact in the prognosis of overall survival (OS) and progression‐free survival (PFS).

**Results:**

A total of 136 patients were included in the study. In the total study population, 84 patients were male (61.8%) and 52 were female (38.2%). Median PFS was nine months (95% confidence interval [CI]: 8.4–9.5 months) and median OS was 12 months (95% CI: 11.3–12.6). The results indicated that the most determining prognostic factors for OS and PFS were cell differentiation measured by immunohistochemical biomarkers, the treatment chosen, and that RECIST was the most significant in the evaluation of patient response to treatment.

**Conclusions:**

Malignant pleural mesothelioma is a cancer with a poor prognosis usually diagnosed at an advanced stage of disease. Our study revealed that the prognostic factors for OS and PS were cell differentiation, the treatment chosen and RECIST.

## Graphical abstract

Malignant pleural mesothelioma is an infrequent neoplasia with a poor prognosis where the majority of patients already have advanced disease at the time of presentation. Mesothelioma is a neoplasia with a long preclinical stage that can span from 15 to 40 years. The median PFS in this study was nine months and median OS was 12 months. The most determining prognostic factors for OS and PFS were cell differentiation measured by immunohistochemical biomarkers, the treatment chosen, with RECIST being the most significant.

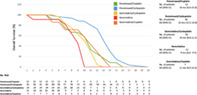

## Introduction

### Epidemiology of pleural mesothelioma in Mexico and the rest of the world

Malignant pleural mesothelioma is an infrequent neoplasm with a poor prognosis and the majority of patients already have advanced disease at the time of diagnosis. In the United States, between two and three thousand new cases are diagnosed every year.^1^ In Western Europe the incidence is higher with almost 5000 new cases per year.^2^

In Mexico, the global dimension of this condition is only partially known as clinical reports are scarce and it is estimated that around 70% of cases are underreported. The approximate number of deaths every year due to mesothelioma is 250 but that figure is probably much higher taking into account the cases which are not reported.

### Asbestos as the main risk factor

Exposure to asbestos is the most important risk factor for malignant pleural mesothelioma. Mexico has multiple asbestos deposits that represent a risk to people who live near to those deposits.^3^, ^4^

The majority of reported series in the literature mention that a history of exposure to asbestos is present in 60%–80% of cases of malignant mesothelioma. In the United States it is estimated that 21 million people working in the construction industry are at risk of asbestos exposure; however, the relatively low incidence of this malignancy makes it essential to search for other etiologies that could predispose the appearance of this neoplasm.^1^, ^2^

The length of time of exposure to asbestos is important because longer periods of exposure increase the burden to the lungs, offsetting the effects of solubility of the fibers and with that it can induce or produce diverse respiratory diseases such as asbestosis, mesothelioma and lung cancer. Mesothelioma is a neoplasia with a long preclinical stage that can span from 15 to 40 years. It has a higher prevalence in males with a male to female ratio of five, and the risk increases with age with a range in diagnosis of 45 to 82 years of age and a median of 72 years.^1^, ^2^

### Treatment of malignant pleural mesothelioma

Malignant pleural mesothelioma is a disease that requires a multidisciplinary approach where different specialties such as Surgery, Medical Oncology, Pulmonary Medicine, Radiotherapy and Pulmonary Rehabilitation among others converge to design a plan for each patient. However, even with this approach, the median overall survival (OS) has been reported to range between nine and 17 months.^2^

### Role of surgery

After initial evaluation, approximately 80% of patients diagnosed with pleural mesothelioma will not be considered candidates for a multidisciplinary approach that includes definitive surgery.^5^

In terms of comparing extrapleural pneumonectomy with pleurotomy/decortication there are no randomized, controlled studies which contrast these two surgical techniques, in the same way there are no controlled studies comparing surgery versus nonsurgical treatment. The current studies are retrospective in nature, have different types of patient cohorts and lack a control group (nonsurgical).

There are several reasons why the surgical treatment of mesothelioma is challenging: (i) Dissemination through the pleural serous surface with early infiltration of the subjacent structures makes a complete surgical resection very challenging;^6^ (ii) the presence of infiltration of the parietal pleural at multiple sites and early compromise of the visceral pleural is very common; (iii) patients tend to be diagnosed late in the disease due to the fact that symptoms are often vague and nonspecific at the beginning;^7^ and (iv) patients are usually adults with associated comorbidities.^6^

### Role of radiotherapy

Radiotherapy on its own is not as effective as primary treatment due to the multifocal nature of the malignancy requiring wide fields of radiation. It is also very difficult to achieve sufficient doses of radiation to accomplish adequate control. Moreover, the required doses are intolerable to the lungs and probably also carry a high cardiac toxicity. The results of chemotherapy in terms of partial response are in the region of 15% to 20%. Complete responses are rare. As a stand‐alone treatment, radiotherapy and chemotherapy have a very limited role in the treatment of mesothelioma, and the best treatment strategy is a combination of these two modalities with surgery.

### Multimodal treatment

A multidisciplinary approach to treatment is based on the evaluation of the extension of the disease, the overall functional status of the patient including his or her cardiopulmonary status, recognition of other comorbidities and the desire or not to pursue an aggressive treatment. We therefore divided patients into two categories.

Surgical candidates: Defined as those who have a surgically resectable disease limited to one hemithorax and have no medical contraindications for surgery. In these patients, a combined modality conducive to a possible cure is embraced, incorporating surgery directed to a complete resection along with chemotherapy and radiotherapy.

Nonsurgical candidates: These are patients where a complete surgical resection is not possible and those who due to other medical conditions such as advanced age, inadequate cardiopulmonary reserve or significant comorbidities cannot undergo a major surgical procedure. In these patients, systemic palliative chemotherapy and treatment directed to control their symptoms are of most benefit and have a positive impact on their quality of life.

### Chemotherapy as part of the multimodal management of mesothelioma

Combined chemotherapy using a regimen of cisplatin plus pemetrexed became the most utilized regimen based on a Phase III study that showed an increase in OS compared to cisplatin therapy alone. Ever since, this has been the standard of therapy in our institution, although the French MAPS study reports data supporting the addition of bevacizumab to this regimen.

In the EMPHACIS trial, 456 patients were treated with cisplatin at standard doses and then randomized to receive either pemetrexed or a placebo every three weeks. The median survival rate was statistically better with the combination (12.1 vs. 9.3 months) as well as the time to progression of the disease (5.7 vs. 3.9 months) and the rate of an objective response (41% vs. 17%). The EMPHACIS trial did not have an arm to assess the effects of maintenance with pemetrexed.^8^

The results of the MAPS study have established the combination of cisplatin‐pemetrexed‐bevacizumab as the accepted standard of therapy in France for patients with mesothelioma. The National Comprehensive Cancer Network (NCCN) guidelines have included this regimen as an option to the standard first line therapy. In this study, after a median 39 months follow‐up, the progression‐free survival (PFS) increased significantly with the addition of bevacizumab in comparison with the combination of cisplatin and pemetrexed (median 9.2 vs. 7.3 months, hazard ratio 0.61 and confidence interval [CI] 95%: 0.50–0.75). Furthermore, the overall global survival increased significantly with the triple therapy (median 18.8 vs. 16.1 months, hazard ratio 0.77; CI 95%: 0.61–0.95).^9,10^

### Evaluation of treatment response

There are several ways to evaluate the clinical benefit of treatment: Rate of response, rate of control of the disease, PFS and OS.

The rate of objective response and PFS have been utilized as parameters of the efficacy of treatment in previous studies. In the studies on malignant pleural mesothelioma there are two systems of radiographic measurement employed through chest computed tomography (CT) scan, Response Evaluation Criteria in Solid Tumors (RECIST) and Modified RECIST. Modified RECIST measures the pleural cortex or the mass thickness perpendicular to the chest wall in two positions and three separate levels in a chest CT. The sum of these six measurements is used to define the response utilizing the RECIST criteria which is one of the variables used in our study to evaluate the response to treatment.^11^

### Prognostic survival factors

One of the most recent series addressing the prognostic factors in mesothelioma was carried out in Taiwan. This series postulated the following variables as significant prognostic factors in patients with advanced pleural mesothelioma: Age, clinical stage, histological subtype and functional status of the patient. Surgical treatment was independently associated with a favorable prognosis in potentially resectable disease (stage I, II and III), whereas systemic chemotherapy was an independent predictor of longer OS in patients with advanced malignant pleural mesothelioma (stage III and IV). This was one of the largest series of cases with this pathology in Asia, outside of Japan. The details of every patient in this study allowed for an integral adjustment of prognostic factors together with other previous studies based in registries of Asian countries.^12^

The male prevalence reached up to 90% of the patients, and the epithelioid histological subtype was present in more than half of the patients with mesothelioma in this retrospective analysis. The frequency of sarcomatoid and biphasic subtypes was reported to be between 10% and 20%.^12^

In the Mexican population there have been only a small number of studies to determine prognostic factors. In one of these, molecular markers such as BCL‐2, p27, MIB‐1 and p53 antibodies, were obtained. The rate of positivity was 2% for the BCL‐2, 61.2% for the p27, 70.5% for the MIB‐1 and 42.1% for the p53. The survival period also showed statistical significance with an increase in the molecular markers and tumor cellular proliferation with the advancement of the disease. A Cox survival analysis did not show a statistical significance when comparing some clinical variables recognized as risk factors. This study suggested that the expression of p27, p53 and MIB‐1 is associated with prognosis in patients with malignant pleural mesothelioma.^13^

## Methods

This was a descriptive, observational, retrospective study of 136 patients with a confirmed diagnosis of mesothelioma, comparing histological subtypes, immunohistochemical biomarkers, concomitant chronic degenerative diseases, tobacco use, age at the time of diagnosis, clinical stage and chemotherapy agents used or other treatments such as radiotherapy and surgery to identify all the factors that impact the prognosis, OS and PFS. The study design was approved by the Instituto Nacional de Enfermedades Respiratorias Institutional Ethics Board following the Declaration of Helsinki, Fortaleza Brazil, 2013. The study population met the following criteria: Patients of 18 years of age or older with a confirmed histopathological diagnosis of mesothelioma, and ECOG 0–3, locally advanced and/or metastatic clinical stage. The following clinical and pathological variables were reviewed: Sex, age, smoking status, asbestos exposure, diagnosis, immunohistochemical biomarkers, treatment used, Eastern Cooperative Oncology Group (ECOG) 0–3, TNM initial clinical stage, response to treatment, PFS and response as per Response Evaluation Criteria in Solid Tumors, version 1.1 (RECIST 1.1) using computed tomography (CT) scan.

### Statistical analysis

SPSS software version 24.0 (IBM, Armonk, NY) was used for analysis. The variables were expressed as median values, together with total values and percentages. PFS and OS were graphed using a Kaplan‐Meier plot. The criterion for statistical significance was *P* < 0.05. All financially related issues from the study were absorbed by the investigation group.

## Results

A total of 136 patients were included in the study. From the total study population, 84 patients were male (61.8%) and 52 were female (38.2%). The average age was 68 years with a range of 41 to 90 years. Median weight at the time of diagnosis was 59.4 kg (range: 43–90 kg). A total of 92 patients (67.7%) had a confirmed history of tobacco use (current or former smoker), 37 (27.2%) had a confirmed history of diabetes mellitus and 62 (45.6%) of systemic arterial hypertension. One patient (0.7%) had an ECOG score of 0, 34 patients (25.0%) an ECOG score of 1, 91 (66.9%) an ECOG score of 2 and 10 patients (7.4%) an ECOG score of 3. Of all 136 patients, 84 (61.8%) were at clinical stage IV, 26 (19.1%) were at clinical stage III, 14 (10.3%) were at clinical stage II and 12 (8.8 were at clinical stage I, according to the eighth edition of the TNM Classification for Lung Cancer provided by the International Association for the Study of Lung Cancer (IASLC).

A total of 136 patients with a diagnosis of mesothelioma were studied, of which 130 (95.6%) had epithelioid histological type, three (2.2%) had sarcomatoid type and three (2.2%) had a biphasic type. Of those histological types, 100 (73.5%) patients had solid subtype, 12 (8.8%) had tubulopapillary, six (4.4%) had acinar subtype, six (4.4%) patients had micropapillary subtype, five (3.7%) had solid papillary subtype, one (0.7%) had tubuloacinar subtype, one (0.7%) had papillary subtype and one (0.7%) had pleomorphic subtype. A total of 93 (68.4%) patients were examined for nuclear calretinin of which 54 (39.7%) were positive (+++), 23 (16.9%) ++, 14 (10.3%) + and two (1.5%) were negative. A total of 92 (67.6%) patients were examined for cytoplasmic calretinin of which 54 (39.7%) were positive (+++), 25 (18.4%) ++, 11 (8.1%) + and two (1.5%) were negative. For nuclear WT1 95 (69.9%) patients were evaluated. A total of 57 (41.9%) were positive +++, 25 (18.4%) were ++, 11 (8.1%) were + and two (1.5%) were negative. Cytoplasmic WT1 was evaluated in three (2.2%) patients and only one (0.7%) was positive (+++). A total of 72 patients (52.9%) were studied for CK‐5/6 and 28 (20.6%) were positive +++, 17 (12.5%) were ++, 17 (12.5%) were + and 10 (7.4%) were negative. CK‐7 was evaluated in 68 patients (50.0%) of which 35 (25.7%) were positive +++, 23 (16.9%) were ++, four (2.9%) were + and six (4.4.%) were negative. As for cytoplasmic HBM1, 65 patients (47.8%) were examined. A total of 35 (25.7%) had +++, 22 (16.2%) had ++, seven (5.1%) had + and one (0.7%) was negative. For BerEp4 15 patients were evaluated (11.0%) of which six (4.4.%) were positive (+++), five (3.7%) were ++ and four (2.9%) were negative. For EMA, 38 patients were studied (27.9%). A total of 23 (16.9%) were positive +++, nine (6.6%) were positive ++, three (2.2%) were positive + and three (2.2%) were negative. A total of 88 (64.7%) patients were evaluated for TTF1 and none were positive. A total of 18 patients (13.2%) were examined for CD‐99, and 11 (8.1%) were positive +++, six (4.4%) were ++ and one (0.7%) was +. For CK20, 15 (11.0%) patients were evaluated, eight (5.9%) were positive +++, six (4.4%) were ++ and one (0.7%) was +. A total of 83 patients (61.0%) were studied for Reβ and 58 (42.6%) were positive +++, 12 (8.8%) were ++ and 13 (9.6%) were +.

Site of metastasis was evaluated and in 109 (80.1%) patients there was no evidence of metastasis, five (3.7%) had metastasis in the lungs, one (0.7%) had metastasis in the central nervous system, four (2.9%) had malignant pleural effusion, seven (5.1%) had metastases in the adrenal glands and 10 (7.4%) had metastases at other sites.

Of all 136 patients, 81 (59.6%) received pemetrexed/carboplatin, 24 (17.6%) patients received gemcitabine/carboplatin, 12 (8.8%) received pemetrexed/cisplatin, 11 (8.1%) received gemcitabine, seven (5.1%) received gemcitabine/cisplatin and one (0.7%) received vinorelbine/cisplatin. The median of cycles received was six (1–13). After evaluating the response to chemotherapy by RECIST 1.1, we found 25 patients (18.4%) with a partial response, 84 (61.8%) with stable disease, 10 (7.4%) with complete response and 17 (12.5%) with progression. In addition, 18 patients (13.2%) received radiotherapy, 19 (14.0%) had surgery of which nine (6.6%) had pleuropneumonectomy and 10 (7.4%) had pleurectomy. There were three (2.2%) patients who had pleurodesis.

**Figure 1:**
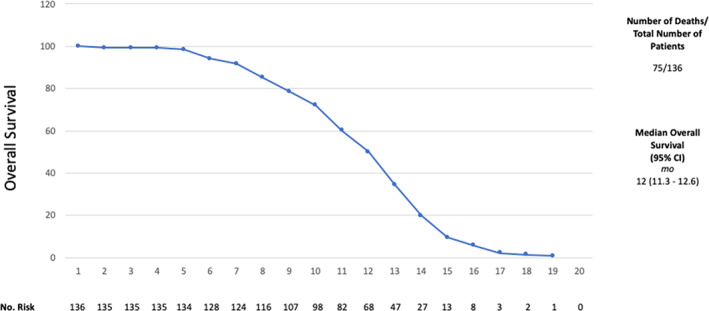
Overall survival (OS).

**Figure 2:**
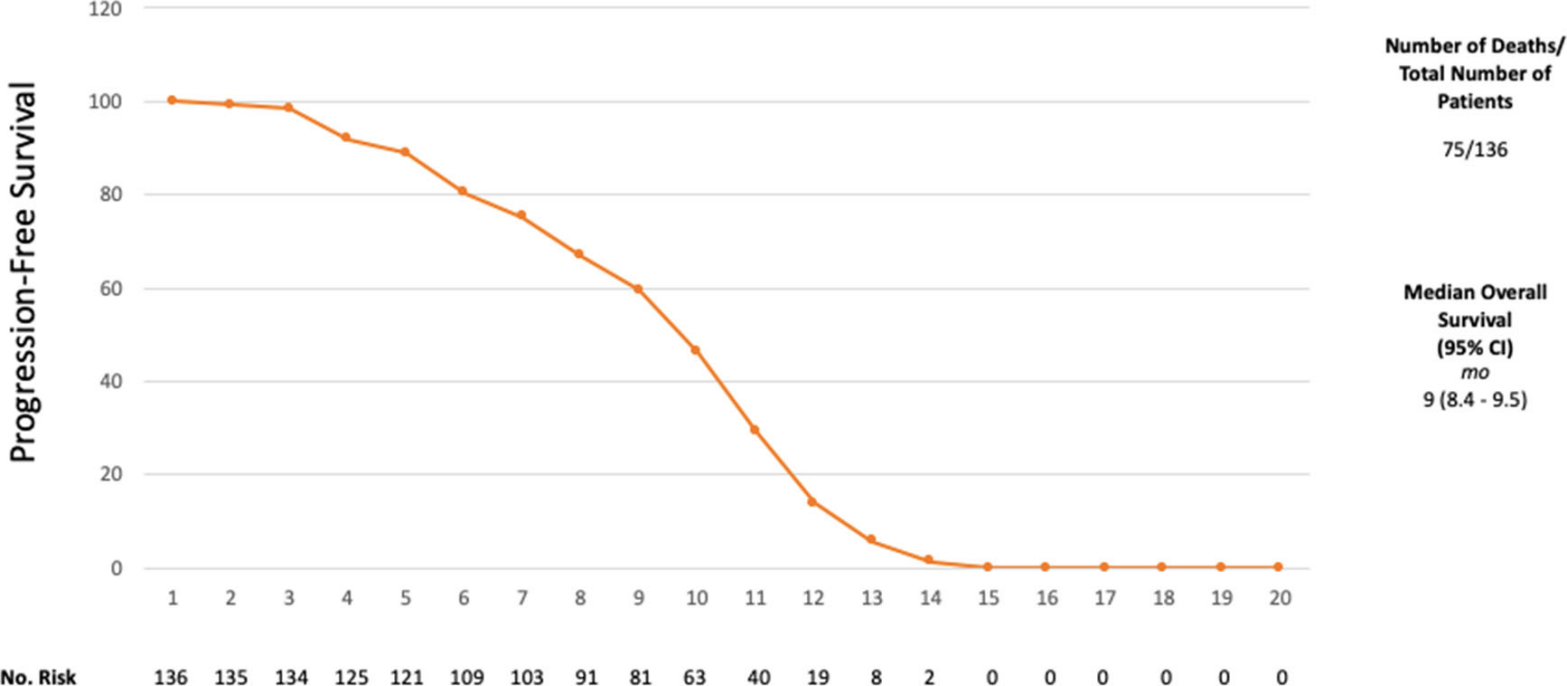
Progression‐free survival (PFS)

**Figure 3:**
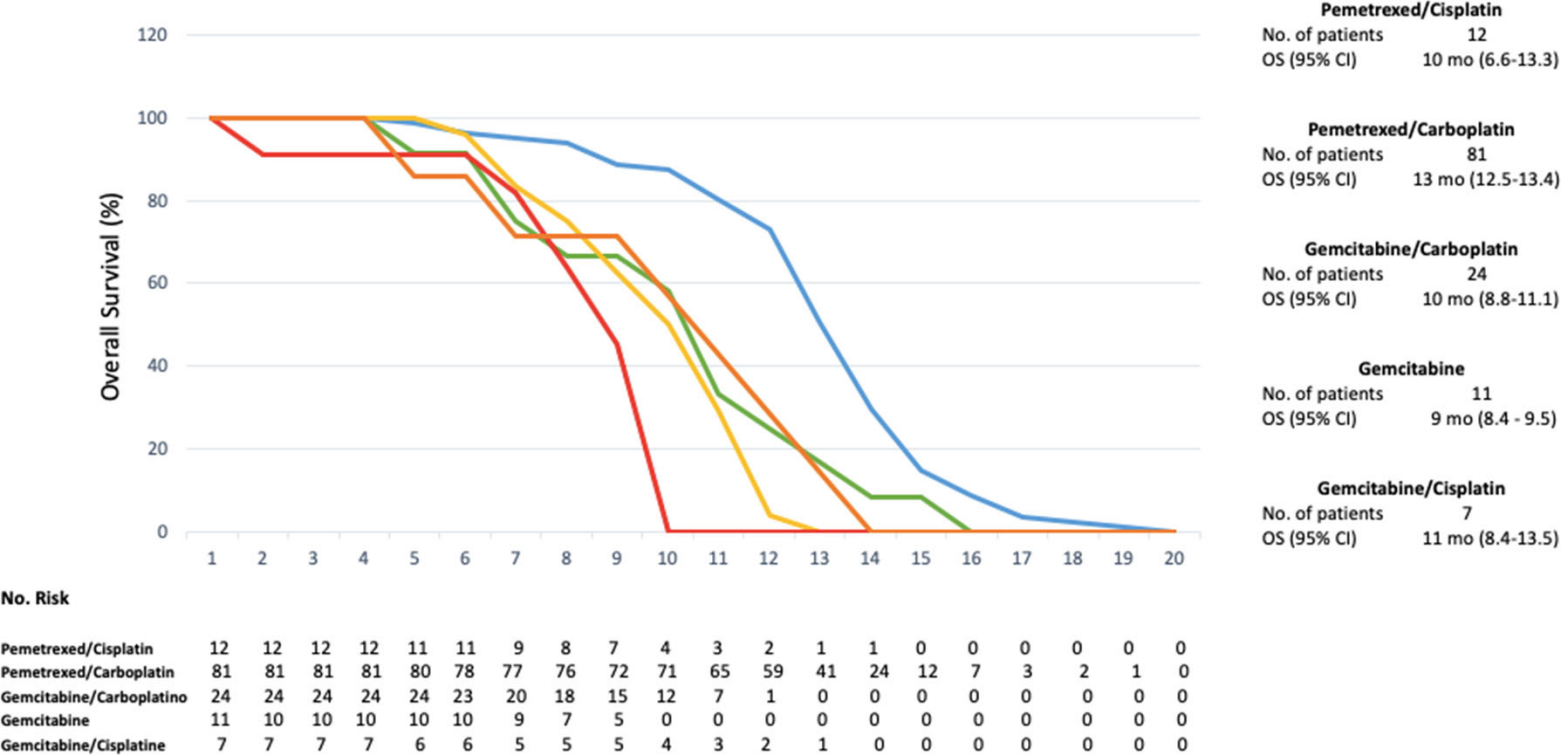
Overall survival (OS) with the chemotherapy regimens used. (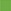) Pemetrexed/Cisplatin, (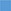) Pemetrexed/Carboplatin, (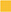) Gemcitabine/Carboplatin, (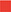) Gemcitabine, (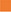) Gemcitabine/Cisplatin.

**Figure 4:**
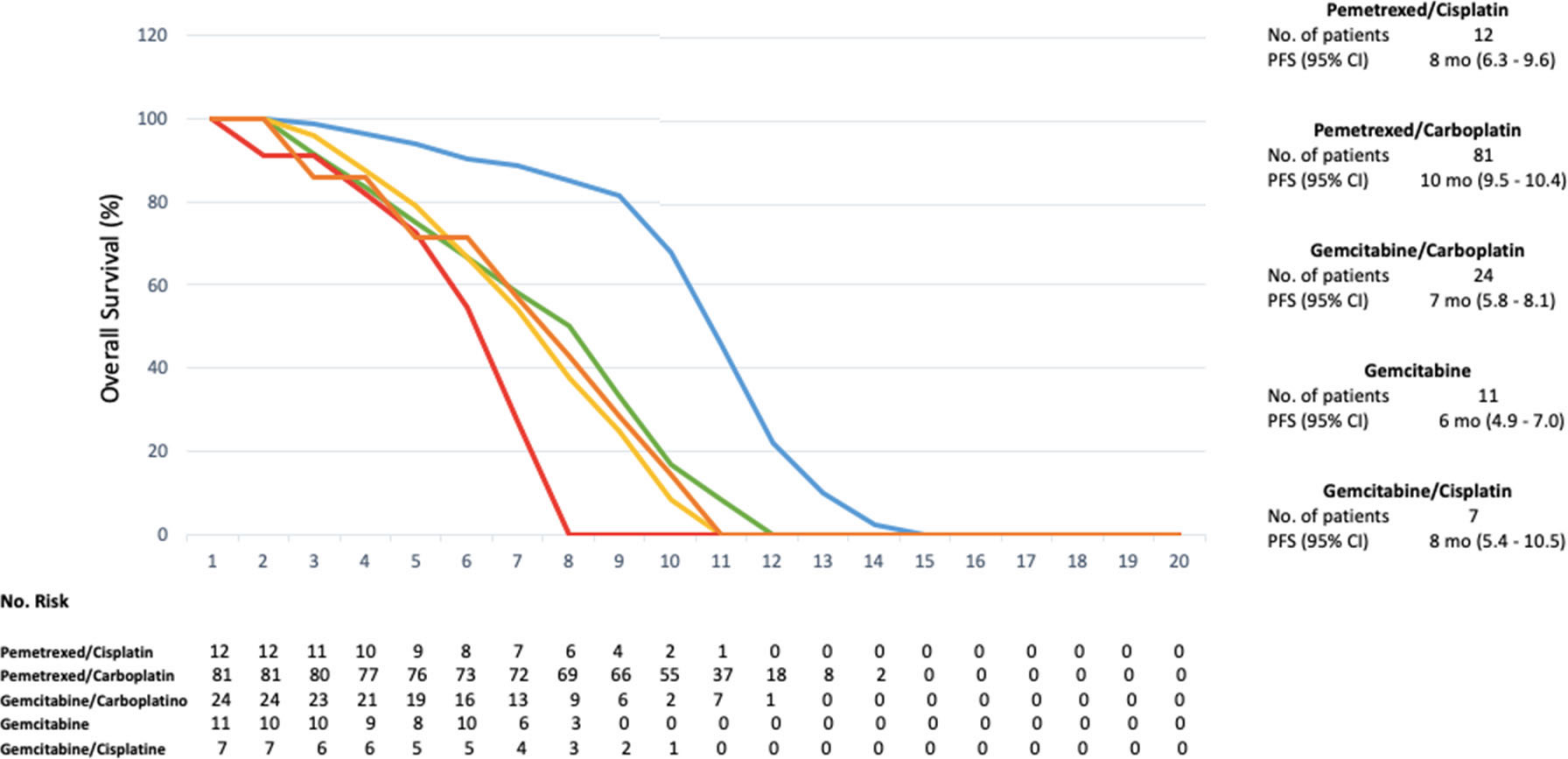
Progression‐free survival (PFS) with the chemotherapy regimens used. (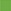) Pemetrexed/Cisplatin, (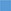) Pemetrexed/Carboplatin, (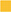) Gemcitabine/Carboplatin, (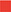) Gemcitabine, (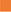) Gemcitabine/Cisplatin.

The median PFS was nine months (95% CI: 8.4 to 9.5 months) and the median OS was 12 months (95% CI: 11.3 to 12.6). Figures 1 and 2. Pemetrexed/carboplatin was the most used regimen in patients with an OS of 13 months (95% CI: 12.5–13.4) and PFS of 10 months (95% CI: 9.5–10.4) (Table 1). Patients who received gemcitabine/carboplatin had an OS of 10 months (95% CI: 8.8–11.1) and a PFS of seven months (95% CI: 5.8–8.1). The use of pemetrexed/cisplatin demonstrated an OS of 10 months (95% CI: 6.6–13.3) with a PFS of eight months (95% CI: 6.3–9.6), gemcitabine alone showed an OS of nine months (95% CI: 8.4–9.5) with a PFS of six months (95% CI 4.9–7.0), gemcitabine/cisplatin had an OS of 11 months with a PFS of eight months (95% CI: 5.4–10.5) while vinorelbine/cisplatin showed an OS of five months and a PFS of three months). Figures 3 and 4.

**Table 1:** Patient characteristics

Characteristic	Patients
Age, years, mean (SD)	68
Sex	
Female	52 (38.2%)
Male	84 (61.8%)
Smoking	
Yes	92 (67.6%)
No	44 (32.4%)
Concomitant disease	
Diabetes mellitus	62 (45.6%)
Systemic arterial hypertension	37 (27.2%)
ECOG	
0	1 (0.7%)
1	34 (25.0%)
2	91 (66.9%)
3	10 (7.4%)
Initial clinical stage	
I	12 (8.8%)
II	14 (10.3%)
III	26 (19.1%)
IV	84 (61.8%)
Histological type	
Epithelioid	130 (95.6%)
Sarcomatoid	3 (2.2%)
Biphasic	3 (2.2%)
Pattern	
Acinar	6 (4.4%)
Micropapillary	6 (4.4%)
Papillary	1 (0.7%)
Pleomorphic	1 (0.7%)
Solid	100 (73.5%)
Solid papillary	5 (3.7%)
Tubuloacinar	1 (0.7%)
Tubulopapillary	12 (8.8%)
Immunohistochemical biomarkers	
Nuclear calretinin	57 (53.3%)
Negative	
+	
++	
+++	
Cytoplasmic calretinin	39 (36.4%)
Negative	
+	
++	
+++	
Nuclear WT‐1	9 (8.4%)
Negative	
+	
++	
+++	
Cytoplasmic WT‐1	2 (1.9%)
Negative	
+++	
CK5‐6	72 (52.9%)
Negative	
+	
++	
+++	
CK7	68 (50.0%)
Negative	
+	
++	
+++	
Nuclear HMBE‐1	0 (0.0%)
Cytoplasmic HMBE‐1	65 (47.8%)
Negative	
+	
++	
+++	
BerEp4	15 (11.0%)
Negative	
+	
++	
+++	
EMA	38 (27.9%)
Negative	
+	
++	
+++	
TTF‐1	88 (64.7%)
Negative	
CD99	18 (13.2%)
Negative	
+	
++	
+++	
CK20	15 (11.0%)
Negative	
+	
++	
+++	
Reβ	83 (61.0%)
Negative	
+	
++	
+++	
Chemotherapy used	
Gemcitabine	11 (8.1%)
Gemcitabine/carboplatin	24 (17.6%)
Gemcitabine/cisplatin	7 (5.1%)
Pemetrexed/carboplatin	81 (59.6%)
Pemetrexed/cisplatin	12 (8.8%)
Vinorelbine/cisplatin	1 (0.7%)
RECIST	
Partial	25 (18.4%)
Complete	10 (7.4%)
Stable disease	84 (61.8%)
Progression	17 (12.5%)
Other treatment used	
Radiotherapy	18 (13.2%)
Surgery	
Pleuropneumonectomy	9 (6.6%)
Pleurectomy/decortication	10 (7.4%)
Pleurodesis	3 (2.2%)

Univariate analysis showed statistical significance in median PFS for nuclear WT1 (*P* = 0.036 and a HR = 0.21), CK7 (*P* = 0.016 and HR = 0.29), patients who received surgery (*P* = 0.001 and HR = 0.66), use of cisplatin versus carboplatin (*P* = 0.005 and HR = 0.25), use of pemetrexed/cisplatin versus other chemotherapy regimens (*P* = 0.001 and HR = −0.35), RECIST (*P* = 0.001 and HR = −0.29) and weight at the time of diagnosis (*P* = 0.002 and HR = −0.26). For OS the variables that demonstrated statistical significance were nuclear calretinin (*P* = 0.024 and HR = 0.23), cytoplasmic calretinin (*P* = 0.033 and HR = 0.22), CK7 (*P* = 0.012 and HR = 0.30), patients who received surgery (*P* = 0.001 and HR = 0.57), the use of cisplatin versus carboplatin (*P* = 0.011 and HR = 0.22), the use of pemetrexed/cisplatin versus other chemotherapy regimens (*P* = 0.001 and HR = −0.34), RECIST (*P* = 0.001 and HR = −0.30) and weight at the time of diagnosis (*P* = 0.002 and HR = −0.26). All these variables were positive in both one‐ and two‐tailed analysis. For PFS one‐tailed analysis, gender, nuclear calretinin, cytoplasmic calretinin and cytoplasmic WT1 also showed statistical significance (*P* = 0.050/HR = 0.14, *P* = 0.027/HR = 0.20, *P* = 0.047/HR = 0.17 and *P* = 0.001, respectively). For OS, one‐tailed analysis, gender was also statistically significant (*P* = 0.026 and HR = 0.16).

## Discussion

Although the use of nivolumab plus ipilimumab as first‐line treatment in unresectable malignant mesothelioma has been recently approved,^14^ pemetrexed since 2003 and platinum‐based chemotherapy association, specifically pemetrexed‐cisplatin, have been considered the gold standard for the nonoperable stages with a median OS of 12.7 months and a PFS of 7.7 months.^15^ In the MAPS study, they demonstrated that the addition of bevacizumab to the standard regimen of chemotherapy improved the OS (18.8 months [95% CI: 15.9–22.6 vs. 16.1 months [95% CI: 14.0–17.9]) and PFS (9.2 months [95% CI: 8.5–10.5] vs. 7.3 months [95% CI: 6.7–8.0]).^9^

In our study, we report the results of patients from a Public Mexican institute whose general characteristics are consistent with previous reports described in the literature. Stage IV was the most prevalent clinical stage in our population, and the average age was 68 years (range: 41–90 years). After evaluation of patients, we found that the median OS in this trial, regardless of the chemotherapy regimen used, was similar to previous studies; 12 months compared with 12.7 months. For pemetrexed/cisplatin the median OS was 10 months compared to 12.1 months in the Vogelzang Phase III study.^16^ For pemetrexed/carboplatin the median OS was 13 months in our study, compared to 12.7 months in the Ceresoli Phase II study.^17^ In this study, the median OS for patients with gemcitabine/carboplatin was 10 months versus 16.5 months in the Favaretto Phase II study.^18^ gemcitabine as monotherapy had a median OS of nine months in our study and 13.1 months in the Mutlu multicenter retrospective study.^19^ For gemcitabine/cisplatin in our study, the median OS was 11 months compared to the Byrne phase II study that was 10.2 months. We only had one patient who received vinorelbine/cisplatin and had an OS of five months compared to the median OS of 8.5 months in the Muers multicentre trial.^20^

To evaluate the objective response, we used the RECIST 1.1 criteria. Independently of the chemotherapy regimen administered, 18.4% had a partial response, 7.4% had a complete response, 61.8% had stable disease and 12.5% had progression which was similar to the results reported in previous studies.

Of all the immunohistochemical biomarkers, those which had an impact on prognosis for PFS were nuclear WT1 (*P* = 0.036 and a HR = 0.21) and CK7 (*P* = 0.016 and HR = 0.29). For OS, nuclear calretinin (*P* = 0.024 and HR = 0.23), cytoplasmic calretinin (*P* = 0.033 and HR = 0.22) and CK7 (*P* = 0.012 and HR = 0.30) showed statistical significance.

For the treatments used in our patients, those who received surgery showed an improvement in PFS and OS with 34% and 43% less risk, respectively (*P* = 0.001 and HR = 0.66, *P* = 0.001 and HR = 0.57). We found a statistically significant difference in the use of cisplatin versus carboplatin, carboplatin being the chemotherapy agent that showed the most benefit in OS and PFS in patients with a 78% and 75% less risk, respectively (*P* = 0.005 and HR = 0.25, *P* = 0.011 and HR = 0.22). For chemotherapy (PFS *P* = 0.001 and HR = −0.35, OS *P* = 0.001 and HR = −0.34), for OS, we found that there was a 66% greater risk of a worse prognosis when it was not received and a faster progression (70% more risk), therefore, the use of chemotherapy is a protective factor. On the other hand, the lower the response according to RECIST (PFS *P* = 0.001 and HR = −0.29, OS *P* = 0.000 and HR = −0.30), the greater the probability of an unfavorable evolution of the disease (71% and 70%, respectively). Likewise, low weight at the time of diagnosis was found to be a risk factor for disease with a poor prognosis at 74% for both PFS and OS (*P* = 0.002, HR = −0.26 and *P* = 0.002, HR = −0.26, respectively). For the multivariate analysis of PFS, the same statistically significant variables were evaluated, RECIST being the only one with a tendency towards significance (*P* = 0.051, HR = −0.54). For OS, the same statistically significant variables from the univariate study were also evaluated, showing RECIST as the only one which was statistically significant (*P* = 0.06, HR = −0.70).

In our study of the Mexican population, the factors associated with a better prognosis for both PFS and OS were the use of the platinum (carboplatin) and pemetrexed regimen, as demonstrated by the EMPHASIS study. Although an extended benefit in the use of bevacizumab in addition to pemetrexed‐cisplatinum has been previously reported in the literature, patients in our country, and specifically in our institution, are unable to afford this treatment. Exclusively for the benefit of PFS, the presence of WT1 and CK7 was associated with a better prognosis and OS for nuclear and cytoplasmic calretinin. With regard to the adverse factors of poor prognosis already known in the biphasic and sarcomatoid subtypes, we found a negative impact of not using the first‐line scheme with carboplatin and pemetrexed, as this combination treatment had the greatest impact on both PFS and OS.

In conclusion, malignant pleural mesothelioma is a cancer with a poor prognosis usually diagnosed at an advanced stage of disease. The most determining prognostic factors for OS and PFS are cell differentiation, measured by immunohistochemical biomarkers, the treatment chosen, and RECIST, this being the most significant.

## Disclosure

The authors declare that there are no conflicts of interest.
